# The Effects of GreenShell Mussel Powder (Brand-Named PERNAULTRA) on Physical Performance and Subjective Pain, Symptoms, and Function Measures in Knee Osteoarthritis: A 6-Mo Randomized, Double-Blind, Placebo-Controlled Trial

**DOI:** 10.1016/j.cdnut.2024.102148

**Published:** 2024-03-27

**Authors:** Cassandra AJ Slade, Marlena C Kruger, Matthew R Miller, Hajar Mazahery, Kathryn L Beck, Cathryn A Conlon, Pamela R von Hurst

**Affiliations:** 1 College of Health, Massey University, Auckland, New Zealand; 2 College of Health, Massey University, Palmerston North, New Zealand; 3 Cawthron Institute, Nelson, New Zealand

**Keywords:** GreenShell mussels, osteoarthritis, KOOS, ICOAP, performance, older adults

## Abstract

**Background:**

Osteoarthritis (OA) can cause disability and reduce quality of life (QoL).

**Objectives:**

This study aimed to determine whether GreenShell mussel (GSM) powder (PERNAULTRA) consumption was more effective than placebo at improving physical performance and subjective measures of symptoms and function in adults with early signs of knee OA.

**Methods:**

The Researching Osteoarthritis and GSM study was a 6-mo randomized, double-blind, placebo-controlled trial in adults aged 55–80 y, screened for signs of OA (*n* = 120, 65.9 ± 6.43 y, 63% female). Participants consumed either 3 g of powdered whole GSM or placebo (pea protein) daily. Baseline and end data collection included 30-s chair stand, stair test, 40-m fast-paced walk test, Knee Injury and Osteoarthritis Outcome Score (KOOS) questionnaire categorized into 5 subscales [pain (P), symptoms except pain (S), function in activities of daily living (ADL), function in sports/recreation (SP), and QoL], a measure of Intermittent and Constant Osteoarthritis Pain, and visual analog scale of pain and symptoms.

**Results:**

Visual analog scale symptoms showed a significantly greater reduction in percentage change for GSM than that for placebo [−28.1 (−59.2, 43.2) compared with 0.00 (−28.6, 100); *P* = 0.03]. Further, a trend for improvement in percentage change for GSM compared with placebo was seen in 40m fast-paced walk [2.51 (−3.55, 8.12) compared with 0.20 (−6.58, 4.92); *P* = 0.09], KOOS-SP [11.4 (−4.48, 27.0) compared with 0.00 (−11.1, 17.7); *P* = 0.09], and Intermittent and Constant Osteoarthritis Pain intermittent pain scale [−27.7 (−77.3, 0.00) compared with −14.6 (−50.0, 36.4); *P* = 0.08]. In those with body mass index (BMI; in kg/m^2^) <25, GSM consumption significantly improved KOOS-S compared with placebo [6.35 (3.49, 12.7) compared with 0.00 (−4.65, 4.49); *P* = 0.03] and showed a trend for improvement in KOOS-ADL [3.29 (1.01, 8.79) compared with 1.01 (−5.75, 4.30); *P* = 0.07]. Those with BMI of ≥25, consuming GSM showed a trend for improvement in KOOS-SP [13.6 (−4.76, 33.3) compared with 0.00 (−12.5, 20.0); *P* = 0.07].

**Conclusions:**

This research suggests consumption of GSM has potential to alleviate symptoms and improve functionality in OA.

This trial was registered at Clinical Trial Registry as ACTRN12620001112954p (https://www.anzctr.org.au/ACTRN12620001112954p.aspx).

## Introduction

Osteoarthritis (OA) is a slow progressive disease incrementally reducing joint function through increasing pain and limiting movement. OA can present in any joint; however, the global prevalence of knee OA in those aged >40 y is estimated at almost 23% [1], and knee OA makes up four-fifths of the worldwide OA burden [2]. OA exhibits a dysregulation in the anabolic and catabolic processes within the joint, causing cartilage breakdown and inflammation leading to symptoms. The primary symptom of OA is pain, which is a major cause of reduced functionality [3]. Other symptoms include joint stiffness, a reduction in range of motion, tenderness, crepitus, and swelling [4]. These symptoms lead to increasing disability, which is detrimental to both physical and mental health, and reduces quality of life (QoL). There are no completely effective treatments. Most conventional therapies target symptom relief, e.g., nonsteroidal anti-inflammatory drugs and analgesics, and the majority have unwanted side effects. Finding therapies that improve OA outcomes by reducing impairment to function and improving symptoms, allowing individuals to increase their number of healthy life years, would be helpful.

OA displays immense heterogeneity. At one end of the scale, an individual can have little radiographic signs of disease and yet experience severe symptoms and disability, and at the other end, radiographically, an individual may show severe OA but experience very few symptoms or debilitating effects. Understanding how potential therapies affect both functionality and symptoms over time is therefore important. Improving function is a critical treatment goal to enhancing QoL [5]. Furthermore, the subjective perception of the individual as to the effects of the disease is key to improving the living reality for those afflicted by OA. Pain and symptoms can be assessed using patient-reported questionnaires such as the Knee Injury and Osteoarthritis Outcome Score (KOOS), measure of Intermittent and Constant Osteoarthritis Pain (ICOAP), and visual analog scale (VAS) of pain and symptoms. Functionality can be assessed using both performance-based and patient-reported measures [6]. The Osteoarthritis Research Society International (OARSI) recommend a core set of 3 performance-based tests to assess functionality in clinical research: the 30-s chair stand test, 40-m fast-paced walk test, and stair-climb test [7]. It is important to measure both self-reported subjective measures of pain, symptoms, and function and performance ability itself because this gives the researcher a thorough understanding of the individual’s functionality. There is evidence of a bias in self-reporting of physical function, having both self-reported and objective measures mitigates this issue [8].

Dietary intervention or supplementation has potential to alleviate OA symptoms by reducing inflammation, protecting against oxidative stress, or increasing the availability of nutrients to repair joint damage. *Perna canaliculus* or GreenShell mussels (GSM) are a native New Zealand shellfish rich in nutrients that could reduce inflammation and improve joint health. These include anti-inflammatory molecules such as long chain omega (ω)-3 PUFAs (predominantly DHA and EPA), antioxidants (eg, vitamins E and C), polyphenols, carotenoids, and joint protective molecules (eg, glycosaminoglycans) [9,10]. Research in humans has found that consuming the lipid component of GSM improves OA symptoms [[11], [12], [13]]. However, only by consuming the whole mussel, can all nutrients that are potentially joint-sparing and beneficial to health enter the body. More recently, research has found whole powdered mussel to benefit those with OA, with those consuming GSM reporting improvements in symptoms [14,15]. Some of the nutrients constituent in GSM are heat and processing sensitive, meaning different powders may possess more potency than others [16,17]. In a recent 12-wk intervention study in women who were postmenopausal and overweight or obese, flash-dried powder of whole GSM meat reduced VAS pain scores to a greater extent than placebo [18]. Further research is needed to investigate the efficacy of this GSM powder in a wider population on improving functionality and symptoms associated with OA.

This study aimed to investigate the effects of 6 mo consumption of whole powdered GSM compared with those of placebo on physical performance measures and subjective measures of pain and symptoms. The hypothesis being that participants consuming GSM would show improvements in the measures compared with those consuming the placebo.

## Methods

The Researching Osteoarthritis and GreenShell Mussels (ROAM) study investigated the effect of GSM intake on patient-reported and performance outcomes in participants with early signs and symptoms of knee OA. The research was approved in November 2020 by the Health and Disability Ethics Committee (ref: 20/CEN/218) and registered in the Australian New Zealand Clinical Trials (registry no: ACTRN12620001112954p).

### Participants

The ROAM study included 120 women and men aged 55–80 y living independently in the Auckland, Northland, and Waikato regions of the North Island of New Zealand. The sample was recruited through voluntary response. The sample size was calculated using KOOS as a primary outcome measure. The power calculation based on a minimally important change of 10 and a SD of 15 [19] specified 47 participants per group were required to detect a clinically significant difference for KOOS with a power of 90%. Exclusion criteria included a history of trauma to knee or hip joints, formal diagnosis of gout or rheumatoid arthritis, allergies to seafood, and regularly taking pain relief medications (more than once per week). Participants were also screened for early signs and symptoms of OA using the KOOS questionnaire where they needed to score of <86 in any of the KOOS subscales to take part in the study. This cutoff has been used in previous research [20]. If eligible, participants undertook a 4-wk washout period from any supplementation that might affect the results, e.g., glucosamine/chondroitin/fish oil, and were not able to take these supplements for the duration of the study. Participants completed a food frequency questionnaire at baseline and end of the study, to ensure that their diet had not changed during the 6-mo trial period. Furthermore, participants were only able to eat oily fish and seafood in accordance with the New Zealand healthy eating guidelines. Weekly questionnaires completed by participants monitored any changes in medications or disease states, which may affect the results.

### Study design

A 6-mo randomized, double-blind, placebo-controlled study design was used. Eligible participants were randomly allocated into 2 groups, intervention or placebo. The intervention group was given GSM powder capsules, and the placebo group received pea protein powder capsules; both groups consumed 6 capsules per day (3 g/d). Participants were asked to consume 2 capsules 3 times daily with meals. Data collection was conducted at the Massey University Nutrition Laboratory in Albany, Auckland, between March 2021 and June 2022. Data were collected at baseline and at the end of the intervention period. Data collection included anthropometry and body composition data (including height, weight, and bioelectrical impedance using the Inbody230 machine) and performance measures and questionnaire data, including health and demographics, physical activity (International Physical Activity Questionnaire) [21], and subjective measures of pain and function (KOOS, ICOAP, and VAS).

### Intervention and placebo

The intervention capsules contained flash-dried whole meat GSM powder (PERNAULTRA; Sanford). GSM composition comprised 41.4% protein, 30.8% carbohydrates, 10.1% fat (20.7% EPA, 8% DHA, 1.1% docosapentaenoic acid), 10.7% ash, and 7% moisture. The placebo capsules contained pea protein powder (Emsland Group) chosen to be approximately comparable with GSM in macronutrient composition, relatively inert, and nonbioactive. The pea protein composition comprised 21.4% protein, 68.9% carbohydrates, 2.6% fat (0% EPA, DHA, and docosapentaenoic acid), 2.8% ash, and 4.3% moisture. Both powders were encapsulated in hard-shell opaque capsules to be visually identical, and activated carbon sachets were placed in bottles to absorb moisture and odours; 3 g/d (equivalent to 1–2 mussels) was given as a realistic amount to consume daily, and both the dose and duration were in line with previous efficacious GSM intervention trials for knee OA [14].

### Randomization

A randomization Excel spreadsheet was completed by an independent researcher not involved in the study. Randomization was stratified based on gender, body mass index (BMI, in kg/m^2^: ≤27 and >27), and age (55–69 and 70–80 y). Bottles of capsules were allocated to the participant, with neither the participant nor the researcher aware of which group the participant was allocated to. All participants and researchers were blinded to treatment group, and only unblinded after all analysis was completed.

### Patient-reported outcome measures

Initially, questionnaire data were collected onsite using computers at the Human Nutrition Unit, but owing to COVID-19 restrictions and the need to reduce participant contact time, all subsequent questionnaires were completed online by participants using links sent via email. Patient-reported outcome measures included KOOS [19], ICOAP [22], and VAS [23]. The KOOS questionnaire data were scored using the KOOS scoring protocol with values ranging from 0 to 100 within each of the 5 subscales, pain (P), symptoms except pain (S), function in activities of daily living (ADL), function in sports/recreation (SP), and QoL. Zero represents extreme knee issues and 100 represents no knee issues [19]. ICOAP data were scored using the ICOAP protocol with values ranging from 0 to 100, with 0 being no pain and 100 being extreme pain, for constant and intermittent pain subscales and for total pain [22]. The first VAS scale asked participants to rate pain in their worst knee on a sliding scale from 0 described as “no pain” to 100 representing “extreme pain.” For the second VAS scale, participants were asked to rate how their knee symptoms were affecting them at the present time on a scale from 0 classed as “not affected by knee symptoms” to 100 classed as “extremely affected.”

### Performance measures

Participants completed the core set of 3 performance-based tests to measure physical function from the OARSI [7]. These were the 30-s chair stand, stair test, and 40-m fast-paced walk test [7]. These are described in detail on the OARSI website [24]. In brief, the 30-s chair stand test measures the maximum number of times the participant can complete a full cycle of moving from sitting to standing and back to sitting again in 30 s. The stair test measures the time taken to ascend and descend a flight of 9 stairs. The 40-m fast-paced walk test measures the time taken to walk 10-m 4 times, excluding turning.

### Compliance and safety

Compliance diaries were emailed weekly to be filled out online. These included questions regarding daily intake of capsules, adverse events, and changes to routines or medications. Compliance was assessed using cumulative capsule counts at the end of the study (number of capsules given at the beginning of the study minus the number of unused capsules returned), and percentage compliance was calculated.

### Statistical analysis

Statistical analyses were performed using IBM SPSS software version 28. The data were assessed for normality using the Kolmogorov–Smirnov test. Normally distributed data are reported as mean ± SD, all other data are reported as median (25th, 75th percentiles) or number (%). The Mann–Whitney *U* test was used for between-group analysis of nonparametric data (not normally distributed). Parametric data were analyzed using independent *t* tests. Results were analyzed both as a total population and stratified by baseline BMI, as previous research has shown significant results in a population with BMI of ≥25 [18]. Statistical significance was considered as *P* value of ≤0.05.

Subjective measures were completed independently by participants (owing to COVID-19 restrictions) allowing room for error. Therefore, participants who had baseline to end percentage change results that were above 2 SDs from the mean for any of the subjective measures were excluded from the analysis: KOOS-S—placebo = 4, GSM = 5; KOOS-P—placebo = 2, GSM = 1; KOOS-ADL—placebo = 2, GSM = 3; KOOS-SP—placebo = 2, GSM = 1; KOOS-QoL—placebo = 4, GSM = 1; ICOAP-Int—placebo = 1, GSM = 1; ICOAP-total—Placebo = 2, GSM = 1; VAS-P—placebo = 3, GSM = 2; and VAS-S—placebo = 4, GSM = 3). This was not completed for performance measures because these were assessed at the visit by the researcher reducing room for error.

As a sensitivity analysis, participants with compliance of <80% (*n* = 5, group A = 2 and group B = 3) were excluded, and the data were reanalyzed. The analyzed results with and without the excluded participants were comparable; therefore, the results of the analysis reported in this study include participants regardless of compliance.

## Results

**FIGURE 1:**
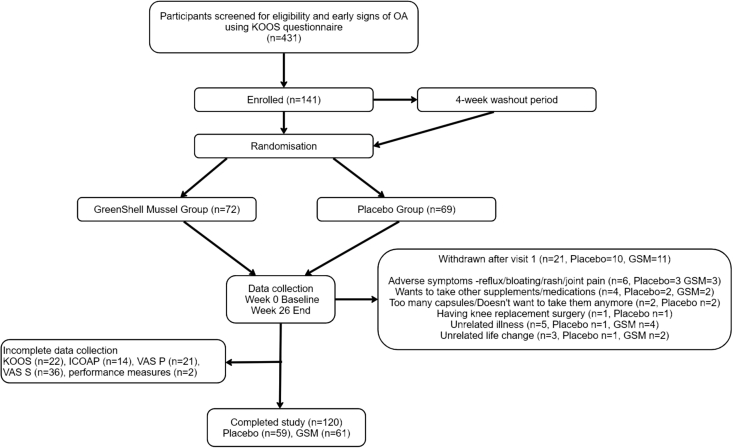
ROAM Study flow diagram. GSM, GreenShell mussel; ICOAP, Intermittent and Constant Osteoarthritis Pain; KOOS, Knee Injury and Osteoarthritis Outcome Score; OA, osteoarthritis; ROAM, Researching Osteoarthritis and GreenShell mussels; VAS, visual analog scale.

Figure 1 displays participant eligibility, enrolment, withdrawal, and completion in the study. Table 1 details demographic characteristics of the study population. In total, 120 participants took part in the study: 59 in the placebo group and 61 in the GSM group. Participants mean age was 65.9 ± 6.43 y, 63% were female, and 85% were of New Zealand European ethnicity. Participant numbers varied across outcome measures owing to noncompletion of questionnaires, inability to complete performance measures due to injury, and removal of outliers. There were no differences in demographics for completers and noncompleters.

**TABLE 1:** Demographic Characteristics by Study Groups.

	Total (*n* = 120)	Placebo (*n* = 59)	GSM (*n* = 61)	*P*_between-group difference_^1^
Age (y)	65.9 ± 6.43	65.9 ± 6.40	65.5 ± 6.37	0.75
Gender				
Female	76 (63)	37 (63)	39 (64)	0.89
Male	44 (37)	22 (37)	22 (36)	
Ethnicity				
NZ European	101 (85)	49 (83)	52 (87)	0.58
Others	18 (15)	10 (17)	8 (13)	
Smoking				
Former and current	23 (19)	17 (29)	6 (10)	0.02
No	96 (81)	42 (71)	54 (90)	
Season of enrolment				
Spring/summer	33 (28)	15 (25)	18 (31)	0.49
Autumn/winter	86 (72)	44 (75)	42 (69)	
Joint supplement use				
Yes	34 (29)	19 (32)	15 (25)	0.38
No	85 (71)	40 (68)	45 (75)	
Anti-inflammatory and antioxidant supplement use				
Yes	36 (30)	20 (34)	16 (27)	0.39
No	83 (70)	39 (66)	44 (73)	
Regular anti-inflammatory medication use				
Yes	2 (2)	0 (2)	2 (4)	—2
No	117 (98)	59 (98)	58 (96)	
Body composition				
BMI (kg/m^2^)	22.6 ± 1.86	28.0 ± 5.92	27.5 ± 6.40	0.52
<25	40 (33)	18 (31)	22 (36)	0.52
≥25	80 (67)	41 (69)	39 (64)	
%BF	26.3 ± 7.89	35.1 ± 9.87	33.2 ± 10.5	0.32
Physical activity				
Low	10 (9)	6 (10)	4 (7)	0.77
Medium	34 (29)	17 (29)	17 (29)	
High	73 (62)	35 (60)	38 (64)	

Values are *n* (%) or mean ± SD.

Abbreviations: BF, body fat; BMI, body mass index; GSM, GreenShell Mussel; NZ, New Zealand.

1 Independent *t* test for normally distributed data, Mann–Whitney *U* test for not normally distributed data, and χ^2^ test for categorical data.

2 Invalid χ^2^ due to high number of cells with expected counts <5.

The impact of the intervention on physical performance measures are summarized in Table 2. No intervention effect was found for the 30-s chair stand test or the stair-climb test. The analysis showed a trend for improvement in percentage change in the 40-m fast-paced walk test for those in the GSM group compared with the placebo group [2.51 (−3.55, 8.12) compared with 0.20 (−6.58, 4.92); *P* = 0.09], respectively. Furthermore, 10 participants in the GSM group improved by over 0.20 m/s (the established minimally important change) [25] compared with only 3 in the placebo group. When the results were stratified by baseline BMI, there was no effect on any of the performance measures.

**TABLE 2:** The Impact of the Intervention on Physical Performance Measures.

	Placebo (*n* = 59)	GSM (*n* = 59)	*P*_between-group difference_^1^
30-s chair stand test (number)			
Baseline	13.0 (11.0, 16.0)	14.0 (11.0, 16.0)	0.28^2^
End point	14.0 (12.0, 17.0)	15.0 (13.0, 18.0)	
%Change	9.09 (−7.69, 20.0)	11.0 (0.00, 27.3)	
Stair-climb test (s)			
Baseline	8.47 (7.25, 10.2)	7.92 (6.78, 9.84)	0.71
End point	8.44 (7.03, 9.60)	7.96 (6.72, 9.41)	
%Change	−7.10 (−16.2, 9.73)	−1.54 (−10.7, 5.81)	
40-m fast-paced walk test (m/s)			
Baseline	1.69 (1.54, 1.93)	1.83 (1.62, 2.03)	0.09
End point	1.74 (1.50, 1.94)	1.81 (1.62, 2.02)	
%Change	0.20 (−6.58, 4.92)	2.51 (−3.55, 8.12)	

Abbreviation: GSM, GreenShell mussel.

1 Independent-sample *t* test.

2 Mann–Whitney *U* test.

**FIGURE 2:**
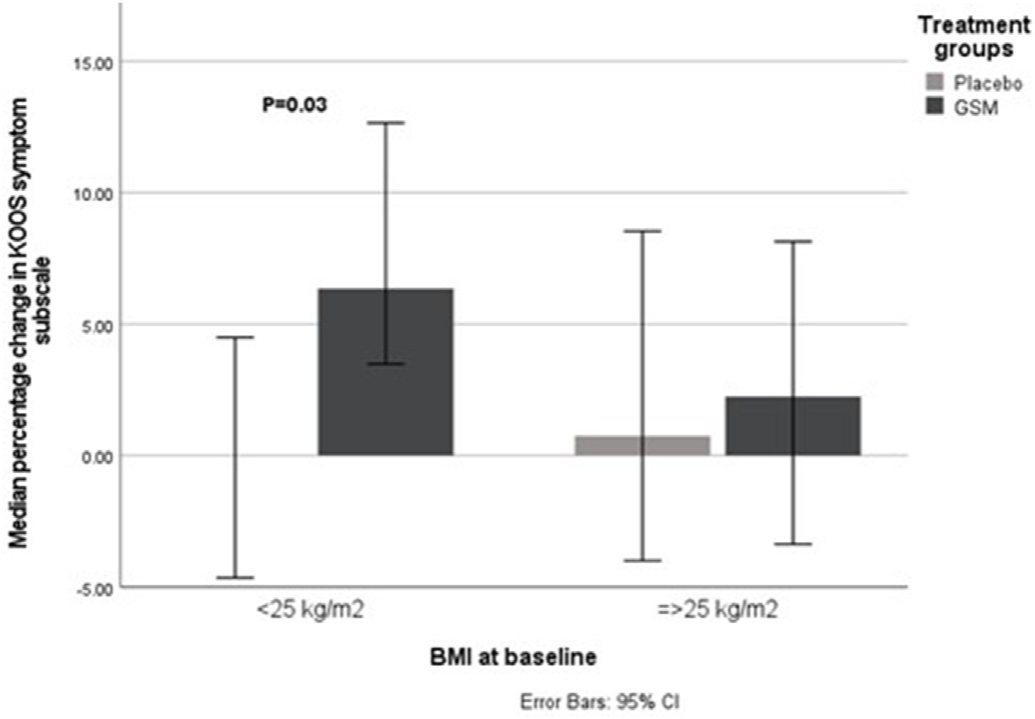
Median percentage change in KOOS symptom subscale in GSM and placebo groups stratified by BMI. BMI, body mass index; GSM, GreenShell mussel; KOOS, Knee Injury and Osteoarthritis Outcome Score.

The impact of the intervention on subjective measures of pain and symptoms are tabulates in Table 3. Those consuming GSM showed a greater improvement in the VAS symptoms scale than placebo (*P* = 0.03). A trend for improvement was also seen in the GSM group for the KOOS-SP subscale and ICOAP intermittent pain. When stratified by baseline BMI, those with a BMI of <25 in the GSM group improved compared with those in the placebo group on the KOOS-S subscale [6.35 (3.49, 12.7) compared with 0.00 (−4.65, 4.49); *P* = 0.03] (Figure 2) and showed a trend for improvement on the KOOS-ADL subscale [3.29 (1.01, 8.79) compared with 1.01 (−5.75, 4.30); *P* = 0.07]. Those with a BMI of ≥25 in the GSM group showed a trend for improvement on the KOOS-SP subscale compared with those in the placebo group [13.6 (−4.76, 33.3) compared with 0.00 (−12.5, 20.0); *P* = 0.07]. Baseline BMI had no effect on response for KOOS-P and QoL subscales, ICOAP, or VAS.

**TABLE 3:** Impact of the Intervention on Subjective Measures of Pain and Symptoms.

	Placebo (*n* = 50)	GSM (*n* = 48)	P_between-group difference_^1^
KOOS			
Symptoms			
Baseline	83.0 (68.0, 93.0)	79.0 (68.0, 88.0)	0.25^2^
End point	86.0 (71.0, 89.0)	82.0 (68.0, 89.0)	
%Change	0.00 (−7.00, 8.86)	4.33 (0.00, 11.6)	
Pain			
Baseline	86.0 (78.0 92.0)	83.0 (72.0, 89.0)	0.63
End point	86.0 (78.0, 94.0)	88.0 (80.0, 93.0)	
%Change	0.00 (−3.00, 10.3)	3.83 (0.00, 9.59)	
Activities of daily living			
Baseline	93.0 (76.0, 99.0)	91.0 (84.0, 96.0)	0.17
End point	93.0 (79.0, 98.0)	94.0 (85.0, 99.0)	
%Change	1.01 (−4.05, 7.53)	3.11 (−1.03, 9.57)	
Sports and recreation			
Baseline	80.0 (60.0, 95.0)	70.0 (48.0, 75.0)	0.09
End point	80.0 (65.0, 90.0)	78.0 (60.0, 95.0)	
%Change	0.00 (−11.1, 17.7)	11.4 (−4.48, 27.0)	
Quality of life			
Baseline	69.0 (56.0, 81.0)	63.0 (50.0, 75.0)	0.92
End point	75.0 (63.0, 81.0)	69.0 (53.0, 71.0)	
%Change	6.82 (−7.95, 17.4)	3.41 (−8.00, 19.1)	
ICOAP (placebo 55, GSM 51)			
Total			
Baseline	11.0 (7.00, 23.0)	11.0 (5.00, 25.0)	0.12
End point	9.00 (5.00, 16.0)	7.00 (0.00, 18.0)	
%Change	−20.0 (−50.0, 41.7)	−33.3 (−71.4, 0.00)	
Intermittent			
Baseline	21.0 (13.0, 25.0)	25.0 (21.0, 28.0)	0.08
End point	17.0 (8.00, 29.0)	8.00 (0.00, 25.0)	
%Change	−14.6 (−50.0, 36.4)	−27.7 (−77.3, 0.00)	
Visual analog scale			
Worse knee pain (placebo 51, GSM 48)			
Baseline	11.0 (0.00, 25.0)	18.0 (2.00, 41.0)	0.59
End point	16.0 (3.00, 29.0)	12.05 (5.00, 43.0)	
%Change	0.00 (−43.2, 150)	−6.23 (−46.8, 60.6)	
Symptoms affect (placebo 42, GSM 42)			
Baseline	7.00 (0.00, 20.0)	22.0 (3.00, 38.0)	0.03
End point	9.00 (1.00, 28.0)	10.0 (1.00, 29.0)	
%Change	0.00 (−28.6, 100)	−28.1 (−59.3, 43.2)	

ICOAP constant pain subscale not included because participants were selected for early signs and symptoms of osteoarthritis and none were in constant pain.

Abbreviations: GSM, GreenShell mussel; KOOS, Knee Injury and Osteoarthritis Outcome Score; ICOAP, Intermittent and Constant Osteoarthritis Pain.

1 Mann–Whitney *U* test.

2 Independent-sample *t* test.

Within the whole population, the number of responders (minimal clinically important change in KOOS subscales ≥10 units [19], ≥−18.5 units for ICOAP [26], and ≥−19.9 units for VAS [27]) did not differ across treatment groups. When the analysis was stratified by baseline BMI, the number of responders in relation to KOOS-ADL was significantly higher in the GSM group than that in the placebo group for those with BMI of <25 [5 (25) compared with 0 (0); *P* = 0.05].

Median compliance for the study was 98.0% (95%, 100%) and similar across treatment groups [placebo: 99.0% (95%, 100%); GSM: 97.0% (95%, 100%); *P* = 0.17]. Seventeen participants who completed the study experienced adverse effects. The most common adverse effect was mild indigestion or reflux experienced by 10 participants (2 in the placebo group and 8 in the GSM group). The remaining adverse effects included a transient change in bowel habits in 5 participants (3 in the placebo group and 2 in the GSM group); nauseous feeling for the first couple of days in 1 participant in the placebo group, which resolved; and feeling intermittently bloated in 1 participant in the placebo group.

## Discussion

The results of this 6-mo randomized, double-blind, placebo-controlled indicate a trend for GSM to improve performance on the 40-m fast-paced walk test. Furthermore, they suggest GSM might be beneficial for symptoms and pain compared with placebo, displaying a trend for improvement on the KOOS-SP subscale and ICOAP intermittent pain scale, and a significant improvement in VAS symptoms score. Those with BMI of <25 in the GSM group also showed a significantly greater improvement than those in the placebo group in the KOOS-S subscale and a trend for improvement in the KOOS-ADL subscale. Those with a BMI of ≥25 consuming GSM, however, showed a trend for improvement in the KOOS-SP subscale compared with those in the placebo group.

GSM consumers showed a trend for improving speed on the 40-m fast-paced walk test, and for 10 participants, this was over the established minimally clinically important difference of 0.20 m/s [25]. This improvement, especially for these participants, was meaningful in reducing the debilitating effects of the disease itself. The results showing no significant difference between groups in performance of the 30-s chair stand test or the stair-climb test may be due to a lack of responsiveness seen for these tests. In a study of participants undergoing total knee arthroplasty, when assessed for responsiveness, defined as the ability of the test to detect change over time in the construct they are assessing, only the 40-m fast-paced walk test was found to be adequately responsive [28]. However, this study also questioned the construct validity of all 3 tests. Furthermore, the inability to improve in sit-to-stand and stair-climb tests may also be because of these tests needing more practice to improve than the walk test, a skill that most people will inadvertently practice every day.

Although many of the results show only trends for improvement in the GSM group, these results are still relevant. Even a small change that eases the burden of disease for the individual has the potential to be meaningful. Subjective measures that include an individual’s perspective of how the disease is affecting them will likely have the most impact on their life. Objective measures may only correlate with certain aspects of the disease, e.g., ADL, missing aspects such as QoL, which will impact how the disease is experienced and therefore how debilitating it is [29]. The trend for improvement in KOOS-SP could be important because OA is associated with an increased likelihood of other comorbidities, e.g., cardiovascular disease [30,31], but increasing the ability to exercise could help toward mitigating this.

The trend for an improvement in the ICOAP intermittent pain score in the GSM group supports other research where nutritional interventions have resulted in improvements in ICOAP pain scores. A strawberry-based beverage was found to improve all ICOAP subscale scores for individuals with OA [32]. It was suggested that this in part could be due to the polyphenols (bioactive compounds also found in GSM) having an analgesic effect. Further support has been shown in a recent 12-wk RCT in women who were postmenopausal and overweight or obese using the same mussel powder, where a larger reduction in VAS pain score was found for those consuming GSM than placebo [18], and a recent systematic review of clinical trials looking at OA and GSM also concluded GSM (whole meat powder or lipid extract) elicited a clinically meaningful benefit in VAS pain scores [33]. Although this study did not show a significant improvement in VAS pain, those in the GSM group did see a negative percentage change in the scale compared with those in the placebo having no percentage change. Furthermore, this nonsignificant result may be due to the VAS assessing general pain in the knee using a visual scale, rather than OA-specific questions as administered in both the ICOAP and KOOS questionnaires. The uniqueness of the experience of pain and the strengths and limitations of the tools used to assess it means it is important to use different tools to ensure all aspects of pain are accounted for [32].

The significant improvement in VAS symptoms score for those in the GSM group supports recent findings from 2 studies in Australian populations that used 3 g/d of a blend of freeze-dried mussel meat stabilized with rosemary oil extract (GlycOmegaPLUS). This showed improvements in symptoms and pain measured by the Western Ontario and McMaster Universities Osteoarthritis Index and Lequesne algofunctional index [14,15]. Symptoms as measured using a VAS is more of a general assessment of symptoms than the specific OA-related questions asked by the KOOS symptoms subscale, and this may be why an improvement was seen in this for the total population but only for the KOOS symptoms subscale in the BMI of <25 group.

The differences in GSM effects for those in different BMI categories could be due to the difference in health status of the participants in these groups. Those with a BMI of ≥25 may have other comorbidities compared with those with a lower BMI, as a higher BMI is linked to increased likelihood of comorbidities [34]. Symptoms related to other comorbidities may affect participant responses to some subscale categories, e.g., ADL. This may explain the improvement seen for the GSM group in the KOOS-S subscale and the trend for the ADL subscale for those with a lower BMI but not for those with a higher BMI and further could explain the number of responders in relation to KOOS-ADL being higher in the BMI of <25 GSM group than that in the placebo group. However, the GSM may have allowed for less uncomfortable exercise for those with a BMI of ≥25, as suggested by the trend to increase for this group in the KOOS-SP subscale. Those with a BMI of <25 may have fewer comorbidities, and their lower BMI may be an indicator that they regularly exercise. This may be the reason no improvement was seen for these participants in the KOOS-SP subscale, as they may already be able to exercise with relative ease, not allowing room for improvement to be seen.

The strengths of this study include its use of whole mussel, giving insight into the effects of the whole food as opposed to just the lipid fraction. Furthermore, the mussel was carefully processed to reduce damage to the active constituents. The length of the trial is also a strength as time is needed to see the effects of nutritional interventions. There are limitations to the study, most notably data collection was ongoing during COVID-19 lockdowns, meaning some of the protocols were compromised, for example, subjective measures were answered online by the participants without a researcher present to clarify interpretation of the questions. The effects of lockdown may have also affected participants’ normal lifestyle patterns. Moreover, analysis stratified by gender was not possible as the cohort was mainly female. The numbers in the male cohort were too small giving analysis a low statistical power. We were also unable to consider an interaction effect of gender as the data were nonparametric. There was, however, an equal distribution of males and females across both treatment groups.

In summary, the results mostly found trends for improvements in the GSM group and therefore should be interpreted with caution. However, the significant findings for improvements in VAS score and in those with BMI of <25, KOOS-S score do suggest taking GSM may provide benefits for those with early signs of OA by alleviating symptoms and improving function. Further research investigating the effects of different doses of GSM would help establish if this is a factor in some of the improvements seen not reaching statistical significance.

## Acknowledgments

We thank Owen Mugridge for his contribution to this research.

## Author contributions

The authors’ responsibilities were as follows –CS: designed research, conducted research, analyzed data, and wrote the paper; MCK, MRM: designed research and provided essential materials; HM: conducted research, analyzed data, and provided essential materials; KLB, CAC: provided critical review of draft and final manuscripts; PRvH: designed research, provided essential materials, and had primary responsibility for final content; and all authors read and approved the final manuscript.

## Conflict of interest

The authors report no conflicts of interest.

## Funding

This project was funded by High Value Nutrition and Sanford Limited. The sponsors were not involved in the study design, collection, analysis, and interpretation of data or writing of the report. There were no restrictions regarding publication.

## Data availability

Data cannot be shared as ethical approval has not been received.
